# An Angle Compared Index with Hybrid of Changes in the Ratio and Amplitude for Quantitative Evaluation of Disease Risk, Biological Function, and Biomarker Efficacy

**DOI:** 10.1155/2019/8693719

**Published:** 2019-08-05

**Authors:** Jin Xiaojun, Liu Hui

**Affiliations:** College of Medical Laboratory, Dalian Medical University, Dalian 116044, China

## Abstract

**Objective:**

The purpose of this study was to describe variations of different cardinal frequency by using angle compared index (ACI).

**Methods:**

The basic principle of the analysis model is to comprehensively consider changes in both the ratio and absolute value as follows: ACI = arctan {Pd⁎(1-Pc) / [Pc⁎(1-Pd)]} + arctan (Pd - Pc) - 45, where Pd represent percentage of disease occurrence in disease group and Pc represent that in control group. The range of ACI was (0~90). Thus, ACIs from different cardinal frequency are comparable.

**Results:**

When biomarkers with similar ratio value, absolute value, or ACI but different positive frequencies were combined, although three indexes (ratio value, absolute value, or ACI) increased after two single biomarkers were combined, only ACI increased with similar amplitudes after two single biomarkers with the same ACI at different positive frequencies were combined.

**Conclusion:**

The ACI provides a better understanding power of biomarker and may be a relatively good index for evaluating the complex events represented by different cardinal frequency from new systems.

## 1. Introduction

Quantitative variations in a particular event are normally described in terms of changes in ratio and absolute values, although these factors are not comparable. For example, resolving power of biomarker is normally described in terms of changes in ratio (odds ratio)[1–3] and absolute values (Youden index)[4–6]. When the cardinal number (value in control group) is relatively small, the increase in ratio may be very high although the absolute increase may be not high. In contrast, when the cardinal number is relatively large, the increase in ratio is not high, but the absolute increase may be highly significant. If the occurrence probability of a biomarker is 0.2 in the disease group and 0.05 in the control group, its odds ratio is 4.75 [(0.2*∗*0.95) / (0.05*∗*0.8)], while its absolute value is 0.15 (0.2 - 0.05). If the occurrence probability of another biomarker is 0.3 in the disease group and 0.1 in the control group, its ratio is 3.86 [(0.3*∗*0.9) / (0.1*∗*0.7)], while its absolute value equals 0.2 (0.3 - 0.1). Therefore, if the ratio is taken as the indicator, the resolving power of the first biomarker is better; however, if the amplitude score is taken as the indicator, the resolving power of the latter biomarker is better. The problem is that the ratio method only reflects changes in the occurrence probabilities of the two groups over time, while the difference method solely reflects the change in amplitude of the occurrence probabilities of the two groups. This represents a serious problem for the comparison of variations in the quantity of events described by different cardinal numbers. Therefore, new methods and indices are required for these purposes. At present, few research reports have addressed this problem. Here, we propose a new biomarker index (angle compared index, ACI) that has the potential to solve the above problem.

## 2. Methods

### 2.1. Analysis Model

**Figure 1:**
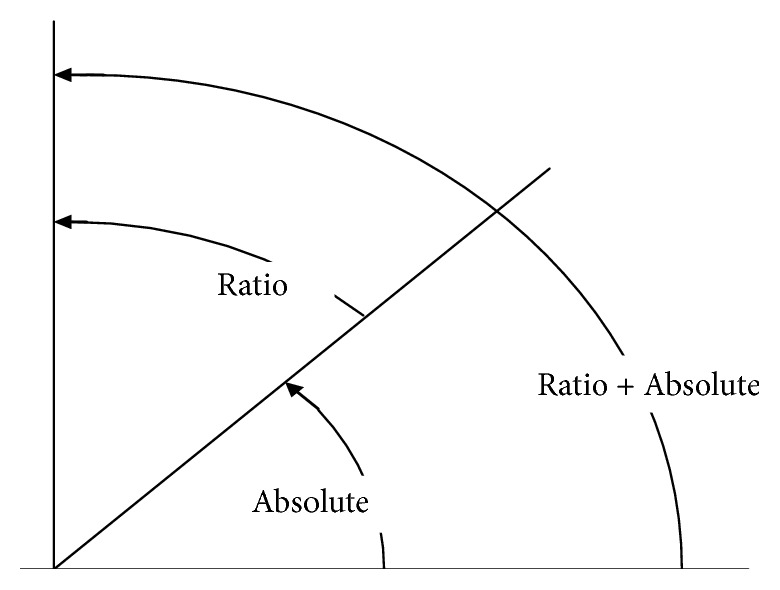
The analysis model for angle compared index.

The basic principle of the analysis model is to comprehensively consider changes in both the ratio and absolute value (hybrid method). We propose a basic principle of the analysis model as Figure 1.

The odds ratio (1 ~ ∞) and Youden index (0 ~ 1) should firstly be translated into angle values as follows according to Figure 1.

New odds ratio = arctan (odds ratio).

New Youden index = arctan (Youden index).

Thus, sum of ratio angle and absolute angle was 45~135; sum range should be transformed into (0~90) as follows and this sum angle was defined as angle compared index (ACI).

(1) ACI=arctan  Youden  index+arctan  odds  ratio−45ACI=arctanPd−Pc+arctanPd∗1−PcPc∗1−Pd−45

where Pd represent percentage of biomarker occurrence in disease group and Pc represent that in control group.

ACI range was (0~90) and can be understood as a amplitude between 0° and 90° as shown in Figure 1, which implies that ACI from different cardinal numbers were comparable. A larger ACI implied a stronger power for a biological function, when ACI was 45, implying a medium effect for a biological factor.

**Figure 2:**
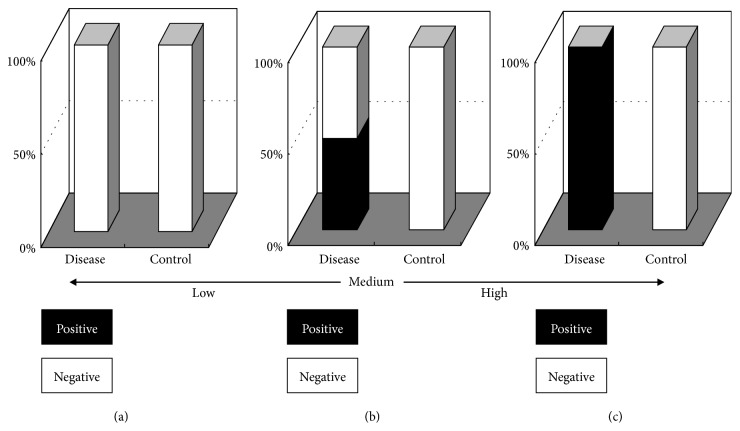
The model for quantitative evaluation of effect strength.

We also consider Figure 2(b) with 0.5 in the disease group (Pd) and 0 in the control group (Pc) as medium effect; ACI should be calculated as 71.5 for Figure 2(b), whereby we suggested that ACI with 45~70 could be defined as moderate effect, ACI with > 70 was could be defined as higher effect, ACI with 45~20 could be defined as lower effect and, ACI with < 20 could be considered as micro effect.

### 2.2. Comparison of ACI with Odds Ratio and Youden Index

A combination of two or more similar efficacy biomarkers may provide more significant resolving power. We used this principle to judge which index of biomarker is better. Two simulated data groups were selected as the disease and control groups depending on design. The simulated data (with 1 and 0 representing positive and negative) was established on the SPSS platform according to random distribution. The frequencies of each group were 0.05, 0.20, 0.30, etc., with each group including 1000 cases (n=1000) and two items (biomarkers). The joint action of multiple indices was evaluated with binary logistic regression [7–9] using the SPSS statistical software package.

### 2.3. Evaluation of Biological Function

The complement tolerance test was done. For example, ten human serum samples were randomly selected and tested for complement activity (C) at standard temperature 37°C (STD.temp) and experimental temperature 47°C (Exp.temp), respectively. The detection method of complement activity was described according to Dong R and Liu H, 2016 [10], in which the complement activity was expressed as C and the rate of Changing (R) was calculated according to the formula:

(2) $$\begin{array}{c} R=\frac{\left(C_{STD\text{.}temp}-C_{Exp\text{.}temp}\right)}{C_{STD\text{.}temp}}, \end{array}$$

A greater R indicated more sensitivity of the complement to the heat.

Regarding complement activity is a continuous variable; the highest value of the complement activity in 10 specimens was used as the denominator. Therefore, we converted it from 0 to 1. Then the converted values were used to calculate ACI. The larger ACI indicated more sensitivity of the complement to the heat.

## 3. Results

Theoretically, as the values are same in disease and control groups, the ACI should give zero score; as the difference between disease and control groups is maximum (1.0), the ACI should give maximum score (90). Our results showed ACI is able to respond these basic facts correctly, suggesting ACI is correct.

When cardinal numbers are very small, the difference (Youden index) should be near 0 for a common biomarker; only ratio value (Odd ratio) contributed to ACI; if ratio values are maximum (*∞*), the ACI could give a half maximum score (45), indicating that ratio weight was equal to absolute change and ACI is reasonable.

It is generally acknowledged that, when contracting the disease of interest is a low-probability event, the related risk (RR) associated with a pathogenic factor in a prospective cohort study is numerically similar to the odds ratio (OR) from a case-control study [1]; therefore, total ACI for special numerus with lower cardinal number was listed in Table 1 for observing the relationship between RR and ACI.

The analysis also indicates when biomarkers with similar Youden index, odds ratio, and ACI but different positive frequencies were combined, all of three indexes were increased; however, the relatively stable score was only obtained for ACI. Detailed results are given in Table 2.

The original data and derived ACI values for the complement tolerance test are shown in Table 3. Specimen 9 was considered as the most sensitive, when R used as an indicator, and specimen 1 was considered as the most sensitive, when used ACI as an indicator.

## 4. Discussion

ACI is the uniform hybrid of changes in both ratio and absolute with same weight; thus, ACI may be more reasonable than RR and Y, which is solely taken ratio or absolute as the indicator for evaluation of disease risk, biological function, or biomarker efficacy. ACI was also considered as an amplitude when the cardinal number was close to zero as Figure 2 and Table 1. Theoretically, as the index increases from 0% to 100%, the increase in amplitude is maximised; the ACI also provided the maximal score at this time (Table 1). When the index does not change or the index is same as before and after the comparison (absolute change is zero), the ACI gave a score of zero. This suggests that ACI accurately reflects the difference in occurrence probability between patients and controls, although ACI is the comprehensive value obtained for index quantity and thus changes over time. The ACI score ranges from 0° to 90°; the significance of quantitative variations in a particular increasing index or risk can be estimated easily according to common-sense judgment.

For another example, for risk factors with RR equal to 10, the morbidity in the exposure group can be relatively high, e.g., approximately 10%, and 1%, but the morbidity may be only 1%, and 0.1%, respectively, in the nonexposure group. Clearly, the risk level of these risk factors is not the same because of different amplitude; the ACI has given different scores (Table 1), suggesting ACI is correct and could provide a better evaluating risk than RR. According to Table 1, ACI could reach 20 when RR is over 2-fold and indicate a lower risk; when RR is over 10-fold, ACI could reach 45 and indicate a medium risk.

A combination of two or more similar efficacy biomarkers may produce more significant resolving power, and the combination of two single biomarkers with the same resolving power but different positive frequencies should provide an equivalent increase in resolving power. Our results showed that only ACI was increased with similar amplitudes after two single biomarkers with the same ACI at different positive frequencies were combined. This indicated that ACI was able to respond to these basic facts correctly and was a relatively better index for evaluating variations in quantity for complex events with different cardinal numbers than Youden index and Odd ratio.

Single nucleotide polymorphism (SNP) is a mainly used genetic marker for medical studies and often represents a small cardinal frequency and a higher ratio (odds ratio)[11–13]. Theoretically, the ACI was around 45 for SNP, implying that SNP was biomarker with lower resolving power according to our suggestion in this study; however, many poorly distinguishing genetic markers may have to be combined to meet the requirements of diagnostic power (ACI >70). It is better those biomarkers screened with relatively near ACI should be combined. We can make a basic evaluation of the resolving power and joint actions of genetic markers with the ACI. The effective genetic markers may be screened with using the ACI.

We can use ACI to explore what extent genetic factors are involved in development of disease. Myopia, for example, myopia rate in children from two groups (parents with or without myopia), was 96.2% and 57.7, respectively [14]; ACI could be calculated as 63.0 for genetic role in myopia and belonged to a moderate effect (ACI > 45).

ACI can also be used to evaluate continuous data. In the complement tolerance test as an example, the complement activity (C) should be first converted to values in the range of 0-1 based on the maximum value; thus, ACI can be calculated. The results of this paper found that in most of the 10 specimens, ACI values were larger than 70, indicating that the effects of the heat on complement have a strong effect. Different people have different complement sensitivity to heat, which may have an important value in diseases research. When R was used as an indicator, Specimen 9 was considered as the most sensitive to heat. And specimen 1 was considered as the most sensitive to heat, when ACI was used as an indicator. Therefore, ACI has a unique significance and deserves in-depth study.

“ACI = 35” can be understood as a amplitude between 0° and 35° as shown in Figure 1, which implies that ACI could be chosen as a common-sense judgment system to intuitively understand the roles of observed factor in complex biological events. Therefore, we propose that ACI provides a further understanding of the quantitative variations in complex events and evaluation of the risks in different cardinal numbers from new concepts with a common-sense judgment system.

## 5. Conclusion

The ACI provides a better understanding power of biomarker and may be a relatively good index for evaluating the complex events represented by different cardinal frequency from new systems.

## Figures and Tables

**Table 1:** Relationship between relative risk (RR) and angle compared index (ACI) for 1% and 0.1% occurrence probabilities in nonexposure group.

1% in nonexposure group			0.1% in nonexposure group			0.1% in nonexposure group		
OP	RR	ACI	OP	RR	ACI	OP	RR	ACI

0.01	1	0.00	0.001	1	0.000	0.200	200	56.033
0.02	2	19.24	0.002	2	18.520	0.300	300	61.521
0.03	3	28.06	0.003	3	26.719	0.400	400	66.674
0.04	4	33.10	0.004	4	31.182	0.500	500	71.470
0.05	5	36.43	0.005	5	33.969	0.600	600	75.892
0.06	6	38.88	0.006	6	35.877	0.700	700	79.938
0.07	7	40.80	0.007	7	37.268	0.800	800	83.620
0.08	8	42.38	0.008	8	38.332	0.900	900	86.959
0.09	9	43.75	0.009	9	39.175	0.999	999	89.953
0.10	10	44.95	0.010	10	39.862	-	-	-
0.20	20	53.45	0.020	20	43.287	-	-	-
0.30	30	59.83	0.030	30	44.814	-	-	-
0.40	40	65.45	0.040	40	45.864	-	-	-
0.50	50	70.53	0.050	50	46.722	-	-	-
0.60	60	75.16	0.060	60	47.485	-	-	-
0.70	70	79.37	0.070	70	48.192	-	-	-
0.80	80	83.17	0.080	80	48.864	-	-	-
0.90	90	86.61	0.090	90	49.513	-	-	-
0.99	99	89.43	0.100	100	50.145	-	-	-

OP: occurrence probabilities in exposure group.

**Table 2:** Combination of two different factors with the same Youden index, Odds ratio, and angle compared index (ACI) but different cardinal numbers.

Index	Marker	Group	One marker		Markers	Two markers (AA' or BB') combined	
			Positive (%)	Index value		Positive (%)	Index value
Youden index	A	Disease	0.20	0.15	A and A'	0.36	0.26
		Control	0.05			0.10	
	B	Disease	0.50	0.15	B and B'	0.75	0.17
		Control	0.35			0.58	

Odds Ratio	A	Disease	0.20	4.75	A and A'	0.36	5.32
		Control	0.05			0.10	
	B	Disease	0.72	4.78	B and B'	0.52	7.94
		Control	0.35			0.12	

ACI	A	Disease	0.20	41.6	A and A'	0.36	49.1
		Control	0.05			0.10	
	B	Disease	0.57	42.2	B and B'	0.82	49.3
		Control	0.30			0.51	

A and A' (or B and B') are two different markers that are distributed at the same frequency in the two groups (disease group and control group) but are independent for each other.

**Table 3:** Angle compared index (ACI) for complement tolerance test.

Specimen Number	Original values			Converted values		
	C_STD.temp_	C_Exp.temp_	R	C_STD.temp_	C_Exp.temp_	ACI
1	0.373	0.063	0.831	0.999	0.169	84.684
2	0.338	0.058	0.828	0.906	0.155	80.802
3	0.349	0.065	0.814	0.936	0.174	81.454
4	0.324	0.060	0.815	0.869	0.161	78.629
5	0.290	0.063	0.783	0.777	0.169	72.995
6	0.368	0.063	0.829	0.987	0.169	84.114
7	0.278	0.057	0.795	0.745	0.153	72.119
8	0.266	0.063	0.763	0.713	0.169	68.883
9	0.317	0.047	0.852	0.850	0.126	79.440
10	0.369	0.081	0.780	0.989	0.217	82.500

## Data Availability

The data used to support the findings of this study are included within the article.
